# Changes of Air Quality During the Pandemic and Aerosol Transmission Issues

**DOI:** 10.1093/nsr/nwaa275

**Published:** 2020-11-24

**Authors:** Weijie Zhao

## Abstract

The COVID-19 pandemic has killed more than 1,000,000 people within nine months in 2020. The world is changed as the cities were locked down, the traffic reduced, and people forced to work from home and keep social distance. These controlling measures also resulted in drastic reduction of the emission of many air pollutants, providing researchers an unprecedented large-scale natural experiment in examining how the air quality would respond to a strong forcing. In this panel discussion held on 22^nd^ September 2020, five experts gathered to discuss their observations and analyses, as well as the current understanding and misconception about aerosol transmission.

This Forum article is dedicated to Prof. Martin Williams of the Imperial College London, who intended to join the panel discussion but passed away one day before it.

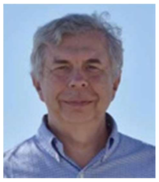

**Guy Brasseur**
Professor of Max Planck Institute for Meteorology, Germany

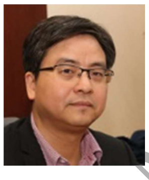

**Junji Cao**
Professor of Institute of Earth Environment, Chinese Academy of Sciences, China

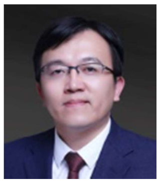

**Aijun Ding**
Dean and Professor of School of Atmospheric Sciences, Nanjing University, China

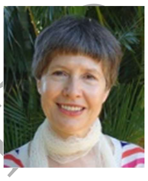

**Lidia Morawska**
Professor of Queensland University of Technology, Australia

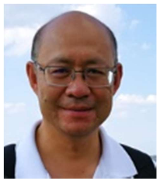

**Tong Zhu**
Dean and Professor of College of Environmental Sciences and Engineering, Peking University, China

## Haze in Beijing during the lockdown? Why?

**Zhu:** Since the outbreak of the pandemic, many countries imposed multiple restrictions, which greatly changed our life styles and the air pollutant emission patterns. How did the air pollution changed during the past several months? What were your observations?

ORIGINAL UNEDITED MANUSCRIPT

**Brasseur:** We have a number of ways to trace how the air quality has been changing, including the satellites and the ground stations. The lockdown started first in China around 23rd January 2020, when China decided to stop a large number of activities. Using TROPOMI (Tropospheric Monitoring Instrument), a payload on the Sentinel-5 Precursor satellite, and other ground-based instruments, we found that the concentration of $NO_2$ in China dropped immediately by 40-50% after the lockdown started [1].

Later in Europe and other regions, a reduction of $NO_x$ was also observed. But the amplitude of this reduction was very different from region to region since the lockdown policies were different. In some countries of Europe, we noticed a large reduction, but in other parts of Europe as well as countries such as the US, the reduction was more limited.

It was interesting to see that in north China, even though the $NO_2$ and PM (particulate matter) level dropped in most places, there were some exceptions. In some regions, including the city of Beijing, one observed peaks of ozone and PM in early February. What were the reasons for these unexpected observed values? Were the enhanced PMs primary PMs (emitted at the surface) or secondary PMs (produced in the atmosphere by some chemical mechanisms)?

**Zhu:** Yes, it was very surprising during the early February when we saw the peaks of $PM_{2.5}$. There were people asking why we had PM increased during the pandemic lockdown, and did that mean all the measures we had taken to constrain PM were not useful.

**Ding:** We tried to answer these questions in our recent paper published in *National Science Review* [2]. We collected data on $PM_{2.5}$ chemical compositions from more than 50 Chinese cities and found significant enhancement of sulfate and secondary PM in a number of cities.

Based on these observations, we provided a conceptual model. Firstly, the reduction of $NO_x$ was much more significant than the reduction of VOCs (volatile organic compounds). This resulted in a weakened NO titration effect, which decreased the ozone loss and therefore increased the ozone level. At the same time, the daytime ozone production and the oxidization capacity were enhanced at regional scale and thus increased the level of both daytime OH radical and nighttime $NO_3$ radical. Then, all these enhanced atmospheric oxidization capacity, including increased ozone, OH and $NO_3$ radicals, would enhance the formation of secondary PM. At that time, Beijing and the surrounding areas were downwind of the entire eastern China with enhanced atmospheric oxidization capacity, so the long-range transport of the enhanced secondary PM from the south contributed a lot to the haze appeared there in February.

**Zhu:** In your model, the reduction of $NO_x$ was much faster than that of VOCs. I'm curious about the reason of that.

ORIGINAL UNEDITED MANUSCRIPT

**Ding:** This is based on our emission estimation using a bottom-up method with human activity data. The main reason is a sustainably reduction of transportation emission. This is particularly strong in the megacities of eastern China. However, the reason is different in South China, where the relatively high air temperature and more forests could cause a lot of biogenic VOCs.

**Brasseur:** The $NO_x$ emissions are very much related to the traffic. When we examine a detailed map of emissions, we noticed large values along the roads, but, in the case of VOCs, the emissions were more geographically spread because the sources involved were different. A major VOCs source is industrial solvents, the use of which was not reduced as drastically as the traffic. There are many species and types of VOCs, and their emissions changed differently.

**Cao:** We did a lot of high-resolution source apportionment studies in the city of Xi'an. We found the emission of three major sources dropped a lot, including dust emitted from road and the construction sites, industrial emission and the local emission. But there were also increasing trends for some sources during the COVID period. For example, the emission from residential heating increased because during the spring festival in China, more people stayed at home and some of them burn coal and biomass to heat up their houses.

**Morawska:** Yes - and energy production hasn't reduced much in many places. This may also be one of the reasons that VOC emissions are staying stable without major change.

**Zhu:** Thanks for all these explanations. We can see that the change of the ratio of different chemical emissions can also help to explain different trends of PM and ozone in different places in the world.

## Situations worldwide

**Zhu:** We talked a lot about the situation in China, especially the complicated trends of ozone and PM. Has this trend been observed in other countries?

**Brasseur:** We did saw a similar pattern of ozone in China and Europe. In northern China the ozone has been increasing but in southern China the ozone was generally decreasing, even though there were increases in the areas around Guangzhou and Hong Kong. In Europe, we also saw an increase of ozone in the northern part and a decrease in the southern part, even though the $NO_x$ was decreasing everywhere.

We first thought that the situation of Europe was comparable to what we observed in China two months earlier. We thought that the chemistry was more winter-like in the north with slow photochemical activity, and summer-like in the south with more solar radiation available. We explained the enhanced ozone in northern Europe by reduced

ORIGINAL UNEDITED MANUSCRIPT

titration of this molecule and reduced ozone in southern Europe by reduced ozone photochemical formation in response to the lowered $NO_x$ emissions during the pandemic.

But actually, we also found that these anomalies in Europe were to a large extent related to the particular meteorological situation that happened at that time. During March and April, the north of Europe was full of solar radiation but in the south, particularly in Spain, a lot of clouds were present (with rain) and slowed down the photochemical processes and hence the ozone formation. This was also confirmed by another research group in Spain.

So the point to make is that, when we do analysis, it's very important to take into consideration the particular situation of the meteorological state of the atmosphere.

**Morawska:** I read a lot of papers about COVID and the atmospheric situation as associate editor or reviewer for academic journals. The decrease of $NO_x$ is a global observation. Most papers reported the concentration of certain pollutants and tried to correlate the decrease with the restriction of traffic. But fewer people tried to provide proper analysis of the complex situations concerning ozone and PM, which are to a large extent not directly emitted by the traffic and are impacted by meteorology and other factors.

I think the situations are extremely location specific. The key element is to understand which sources were actually operating and which were not. For example, you talked about residential heating in China, which was not occurring in Australia. It was summer in the southern hemisphere and there was no heating.

The lockdown is also a major difference. Many cities were supposedly in lockdown, but if you look at the arterial roads, there were less people travelling, but the traffic was not totally stopped. Therefore, we should actually understand what sources were operating before we can understand what really happened.

**Zhu:** Yes, the responses are location specific. And as have been mentioned, specific meteorological and other factors may contribute to the different responses in different regions.

**Brasseur:** In China the lockdown took place very early, when it was still winter. And the lockdown in Europe and US took place in spring and summer. We were in different chemical regimes. Actually, if China had started the lockdown for whatever reason in April or May, you would probably not have seen the increase in ozone. You might have seen a decrease in ozone.

We used a global earth system model to assess the response of the atmosphere to the reduced emissions during the pandemic. If we reduce the industrial and traffic emissions, we can simulate the response that was observed in China in January to March 2020.

ORIGINAL UNEDITED MANUSCRIPT

These models also derive a reduction in surface ozone concentrations in most regions of the northern and southern hemispheres later in the season (April to June). We are looking into each of these areas in details.

We should pay much attention to the meteorological situation since there might have been some specific or unusual situations for a couple of weeks or months like the situation we had in Europe. So we need to make regional analysis based on the specific meteorological stations in different parts of the world.

## Lessons from the unique natural experiment

**Zhu:** We got a lot of data in this pandemic, which can be considered as a large-scale natural experiment. We shut down many activities and we saw the responses. So I think another question is: how could the new data help to optimize the current models?

**Brasseur:** I think there are two distinguished types of modeling: forward modeling and inverse modeling.

In this pandemic, the largest unknown factor is that we don't know exactly how much the emissions have been changing. Inverse modeling can help us to investigate this question. We can combine the observed information on the atmospheric chemical compositions with detailed chemical transport models and use inverse modeling to find out by how much the emissions have been decreasing during the lockdown period. Through this top-down approach, we can estimate what happened for each source and how the emissions were affected by the pandemic in different countries. I'm pleased to hear that there has been some works done in China and we are also doing similar work.

On the other hand, we can make an estimation of the changes in the emission during the lockdown and implement them in forward models to try to reproduce the observed concentration of ozone, PM and other chemical species. In that way, we can test the mechanisms included in our models. If we want to test our model, we have to really push it very strongly into a situation that is far away from what is considered to be a normal or a usual situation. If, under these circumstances, the model is able to represent reality, then we can trust that the model is doing a good job. But if our model does not represent reality, which is the most interesting case, then we can start asking ourselves: what have we missed? What is the process that we have not included in our model? And this is how science is progressing. The large experiment that we are witnessing today is a unique opportunity to push our model in a rather extreme situation that otherwise will never exist, or probably will never exist again unless we have another pandemic in the future.

**Ding:** In our modeling works, we found that the model performed very well in the simulation of ozone and nitrate, but it is still very difficult to reproduce the changes of

ORIGINAL UNEDITED MANUSCRIPT

sulfate and SOA (secondary organic aerosol) in China. It has been a difficulty for years and I think there may be some factors missing. For example, maybe we neglected the primary emission or the fast formation of sulfate from power plants, and maybe we have not fully understood the generation mechanism of SOA or some new mechanisms findings haven't been updated in the models yet. There is still a lot of work to do to improve the models.

**Zhu:** Thanks for your comments. The pandemic offered us a unique natural opportunity to test and improve the models. Some people may want to know: are we now able to predict the future trends of air quality using forward modeling?

**Brasseur:** We probably cannot predict what's going to happen, because we do not know the input, we do not know how people will behave in the future. However, we can do some projection under some possible scenarios. For example, if the pandemic lasts for another year, or two or even three, we can predict how our environmental system or the atmosphere would be responding to estimated changes in emissions.

## Pollution transmission? No! Aerosol transmission? Yes!

**Zhu:** Let's move on to the next topic. There have been publications debating about whether or not air pollution may enhance the spread of virus. What's your opinion about that?

**Morawska:** This was an extremely controversial topic. At the beginning of the pandemic, there were many publications linking air quality with the spread of the virus and claiming that poor air quality greatly exacerbated the pandemic. But most of these studies were making simple associations without looking into what the causes were. These papers were highly contested and some of them were actually withdrawn.

Actually, outdoor air pollution particles are extremely unlikely to be virus carriers, because the pollution particles are much smaller than the virus vehicles. However, it has been pointed out that in regions where air quality is bad, people are already more susceptible to infection by the virus. So it's not about the spread, but the susceptibility. I guess that's the main summary of the relationship between the two parameters.

**Cao:** I agree with your comments. Generally, poor air quality negatively impacts human health and enhances virus infection. High concentrations of PM, $SO_2$ and $NO_x$ are all harmful to human health. But ozone may be an exception. We had a joint study with Wuhan Institute of Virology, Chinese Academy of Sciences and found that ozone molecules can kill the virus in air in certain conditions. So when we talk about air quality, we need to differentiate different indexes.

ORIGINAL UNEDITED MANUSCRIPT

There are also studies showing that air quality or air conditions, such as air temperature and humidity, can influence the residence time of the viruses in the air. The effects of these factors are complex and some studies gave controversy results. Anyway, we should take these factors into consideration and try to further validate their influences.

**Brasseur:** Morawska said that air particles are unlikely to be virus carriers. Does it mean that if I enter a room after an infected person left, there would not be viruses floating in the air and that I am unlikely to be infected?

**Morawska:** No. What you are talking about is aerosol transmission or airborne transmission, which has been proven to be a major transmission mode of SARS-Cov-2. But in these cases, the virus-carrying aerosol particles are generated from the respiration of the patients, not the outdoor air pollution particles that I was talking about.

**Zhu:** Your explanations are very important to clarify the confusions. I have a question about aerosol transmission. Many studies discovered virus-carrying aerosols in hospitals, but do we have robust evidence to say that these aerosol particles contain enough viable viruses to transmit disease?

**Morawska:** It is a kind of chicken and egg problem. The hospitals are often well ventilated, so the virus concentration measured there are not necessarily very high despite the presence of the patients. In some other studies, researchers did retrospective analysis to measure the virus concentration where there had been an outbreak. But we were unable to predict the outbreaks and measure the exact concentration when the transmission was actually happening.

However, there have been quite a number of studies demonstrating the presence of viable viruses. A number of models and analyses of the outbreaks also strongly indicate that airborne transmission is the most logical explanation.

**Zhu:** Will the virus be killed when the water within the particle evaporates and the salt concentration rises?

**Morawska:** There has been much discussion about this. Actually, this process of evaporation happens extremely fast - within a fraction of a second. So if evaporation were able to kill the virus, airborne transmission would be almost impossible. So the fact that the infections did occur means that the virus can cope with this increased salinity.

**Zhu:** What about the outdoor possibility of airborne transmission? What would be the outdoor safe distance?

**Morawska:** In general, the risk outdoors is significantly lower unless you are in very close proximity, because dilution occurs very quickly. The concentration of the virus emitted by an infected person would very quickly drop below the level of infectivity. In

ORIGINAL UNEDITED MANUSCRIPT

addition, the virus is much less stable outdoors because of UV radiation and other factors.

**Ding:** Will the weather influence the outdoor infectious possibility? For example fogs or winds?

**Morawska:** The possibility of infection depends on the virus concentration and the amount of time exposed. So I think many factors may contribute, including the wind, the distance between you and the patient and how long you were together. But again, generally speaking, the probability of infection is lower outdoors.

## Wear masks

**Zhu:** To prevent airborne transmission, the most effective measure is to wear masks. But there are different opinions about this. What's your observation about this?

**Morawska:** Masks can't reduce the risk by a hundred percent, but they are really quite effective. However, I think there should be much better education about how to wear masks. Some people just wear them below their noses and some others tend to wear them all the time, which is not necessary. Actually, if you wear a mask walking in the park or on the street, when you later come into a shopping center or a crowded bus, you may get tired of wearing it for so long and you may start to wear it incorrectly - and that would be dangerous.

**Brasseur:** In Germany, we're doing exactly as you suggested. We're usually not wearing masks on the streets, but we put it on immediately before we enter a shop or come close to other people.

But there is the issue of acceptance. The acceptance in China is very high, but in many other countries such as the US, many people feel that their freedom needs to be preserved. The problem has become political. In Germany, in spite of the relatively high acceptance that COVID-19 is a serious health issue, there have been street demonstrations of people stating that the pandemic was invented by the government to restrict their freedom. They claim that COVID-19 is just a big flu and that it is more important to be able to work in the office and to spend evening time with friends in bars. It's truly difficult for reasonable people and for the government officials to convince everybody that COVID-19 is a medical issue. It's not a political issue.

ORIGINAL UNEDITED MANUSCRIPT

## The world is changing

**Zhu:** The pandemic has lasted for more than seven months, and there's no sign of a sudden stop. So the final question is how would the pandemic change our research, our life styles and our society in the future?

**Cao:** I think the pandemic will have a positive impact on the improvement of air quality in the next several years. During the pandemic, both the governments and the public talked more about indoor and outdoor air quality, as well as aerosol and its heath impacts. So in the future, there will be more support for the research of air quality and aerosol. We should take this opportunity to solve the major scientific problems that may benefit our society in the future.

**Morawska:** It's true that we are starting to care more about indoor air quality. We are working towards a paradigm change in that when we design buildings or other enclosed public spaces, we consider how to mitigate the transmission of respiratory diseases.

For the whole of society, whatever happens, this pandemic will be transformative. That's what we can learn from history. The world is never the same after a pandemic. There's no doubt that there will be changes in many different ways. One of the changes clearly emerging is that we will be working much more from home than from offices. We will not go back to the old system. But it's still difficult to predict other changes at this stage. Maybe the frequency of long-distance travelling will decrease, and maybe we will learn about the benefits of reducing energy consumption. Maybe we will try to build cities with less traffic. I know that Paris is creating areas free from traffic. This pandemic may help to encourage this.

**Brasseur:** I agree that it is transformative and a number of changes will be irreversible to a certain extent. But I should say that some of those changes are not due to the pandemic but are, in fact, accelerated by the pandemic. For example, a lot of people were starting to work from home before the pandemic and the pandemic just provides an opportunity to make this happening more rapidly.

On the other hand, people have a short memory and I think that, to a large extent, they will go back to their 'normal life'. Here in Germany, we were in a very low pandemic situation for several months and people are beginning to go back on vacation to the Mediterranean islands. It may be human nature to enjoy social communication and it may be interesting for social scientists to look into how different areas of the world responded differently.

And finally, I would like to say that many years ago we already knew that there will be several virus pandemic episodes in the future, but we did almost nothing to prevent it or at least to be prepared. We just waited and did not do anything. The situation is the

ORIGINAL UNEDITED MANUSCRIPT

same for the climate issue and other grand challenges of our planet. We know that climate change will be a reality and will increasingly affect society in the future. We should learn from this pandemic that long-term concerns are important and we should take actions right now.

**Zhu:** In China, we are also gradually returning to normal. Many people are not wearing masks on the streets and in many occasions. It may be a good sign indicating that we have controlled the pandemic but may also be a risk.

In the future, some of the changes happening will continue to benefit us, for example, working from home and the online conferences or the online-offline hybrid conferences, which is convenient to us in many cases. The pandemic promoted the development of such technologies.

I would like to thank all of you for joining this discussion. This pandemic provided a unique large natural experiment and offered an opportunity to enhance our studies on air quality control and aerosol transmission. We hope that our following research would be able to benefit our society and prepare for the future challenges.

Weijie Zhao is an NSR news editor based in Beijing.
